# A case report of advanced pancreatic cancer patient demonstrating remarkable efficacy with liposomal irinotecan after failure of multiple-line therapies

**DOI:** 10.3389/fonc.2026.1715778

**Published:** 2026-03-25

**Authors:** Siyu Yu, Hong Zhu

**Affiliations:** Department of Medical Oncology, Cancer Center, West China Hospital, Sichuan University, Chengdu, Sichuan, China

**Keywords:** irinotecan resistant, liposomal irinotecan, multiple-line therapy, pancreatic cancer, remarkable efficacy

## Abstract

Pancreatic cancer remains one of the most aggressive malignancies with an exceptionally poor prognosis. We herein report a case of a 53-year-old male diagnosed with pancreatic tail adenocarcinoma accompanied by extensive hepatic metastases. Despite receiving standard first-line FOLFIRINOX chemotherapy, the patient demonstrated progressive disease accompanied by severe anemia and persistently elevated tumor markers. Subsequent second-line therapy combining gemcitabine/nab-paclitaxel (GA regimen) failed to achieve disease control. Third-line treatment employing lenvatinib plus sintilimab (an anti-PD-1 immune checkpoint inhibitor) similarly resulted in disease progression (PD, Progressive Disease). Remarkably, after transitioning to Nal-IRI-based therapy, the patient achieved a durable partial response (PR) sustained for 10 months (Progression-Free Survival, PFS = 10 months) with favorable tolerability. In this challenging case of irinotican-resistant pancreatic cancer involving both hepatic and multiple osseous metastases, Nal-IRI demonstrated exceptional clinical activity as a later line therapeutic intervention, maintaining disease control for nearly one year with manageable toxicity. These observations suggest that Nal-IRI may represent a viable later line treatment strategy for advanced irinotican-resistant pancreatic cancer, warranting further investigation through prospective clinical trials.

## 1 Introduction

Pancreatic cancer is a malignant tumor associated with an extremely poor prognosis, and its incidence is rising globally, posing a significant threat to public health. According to the 2022 Global Cancer Statistics Report, the death toll and mortality rate of pancreatic cancer remain alarmingly high worldwide. In China, the situation is particularly severe, with both the death toll and mortality rate ranking among the highest globally (1). Radical resection (R0) is currently the most effective treatment for pancreatic cancer; however, due to the subtle symptoms of early-stage disease and low rates of regular screening, most patients are diagnosed at an advanced stage who are not suitable for surgery.

For patients with unresectable locally advanced pancreatic cancer or distant metastasis, the selection of first-line chemotherapy is based on the patient’s physical status. Prior to the introduction of liposomal irinotecan in mainland China in April 2022, if pancreatic cancer progressed after first-line chemotherapy, non-overlapping drugs could be chosen for second-line chemotherapy, taking into account the drugs previously administered, as well as the patient’s complications and adverse reactions. Second-line chemotherapy has been shown to be more effective than best supportive care (2). The prognosis for advanced pancreatic cancer remains extremely poor, even with active treatment, the median survival time for patients is often only about 9 to 11 months. The decision to continue chemotherapy in patients with pancreatic cancer who have failed first-line or second-line chemotherapy remains controversial, and there is no established chemotherapy regimen. Here, we present a case report of a patient with adenocarcinoma of the pancreatic tail accompanied by multiple liver metastases and osseous metastases. After disease progression following first-line treatment with the FOLFIRINOX regimen, second-line treatment with GA (gemcitabine + nab-paclitaxel), and third-line treatment with lenvatinib plus sintilimab, the patient achieved a partial response (PR) with liposomal irinotecan. The PR duration persisted for at least 10 months, and the treatment was well-tolerated with minimal side effects.

## 2 Case presentation

A 53-year-old male patient’s physical examination revealed pancreatic mass and multiple liver nodules. There was no nausea, vomiting, abdominal pain or distension. The contrast-enhanced ultrasound of liver confirmed the metastases. Liver biopsy showed adenocarcinoma with necrosis. The tumor biomarkers showed carcinoembryonic antigen (CEA) 86.00ng/ml, serum carbohydrate antigen 19-9 (CA199) >1000.00U/ml, Serum carbohydrate antigen 125 (CA125) 37.60U/ml. Based on the above information, the patient was finally diagnosed with pancreatic tail adenocarcinoma with multiple liver metastases (cT4NxM1 stage IV). The usual first-line treatment options for pancreatic cancer include AG (gemcitabine + albumin-bound paclitaxel), FOLFIRINOX regimen (oxaliplatin, irinotecan, fluorouracil, and leucovorin) chemotherapy. Considering that the patient’s physical condition is good, the mFOLFIRINOX regimen is adopted.

**Figure 1:**
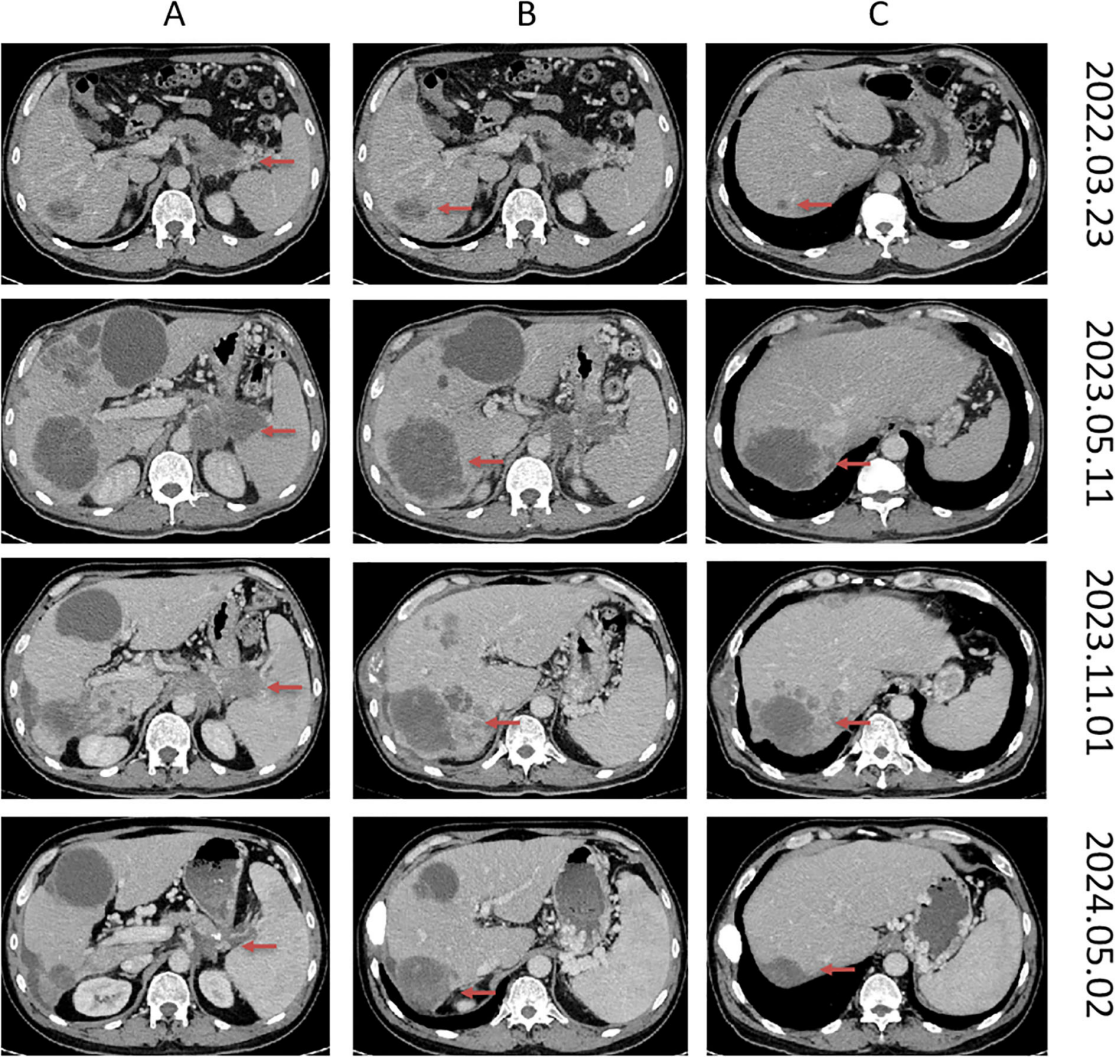
Typical CT images of pancreatic tail cancer and liver metastases before and after treatment at various stages. The red arrow points to the tumor. March 23, 2022 imaging revealed baseline pancreatic cancer lesions along with two liver lesions. May 11, 2023 imaging showed disease progression (PD), which occurred after completion of 13 cycles of FOLFIRINOX chemotherapy. During the treatment course, the patient received maintenance therapy with S-1 for a period, and progression was documented following the final FOLFIRINOX treatment. The November 1, 2023 scan was obtained prior to initiation of liposomal irinotecan plus fluorouracil therapy. The May 2, 2024 scan was obtained after six months of treatment with liposomal irinotecan plus fluorouracil. **(A)** demonstrates changes in the primary pancreatic lesion before and after treatment. **(B, C)** represent changes in liver metastatic lesions before and after treatment.

**Figure 2:**
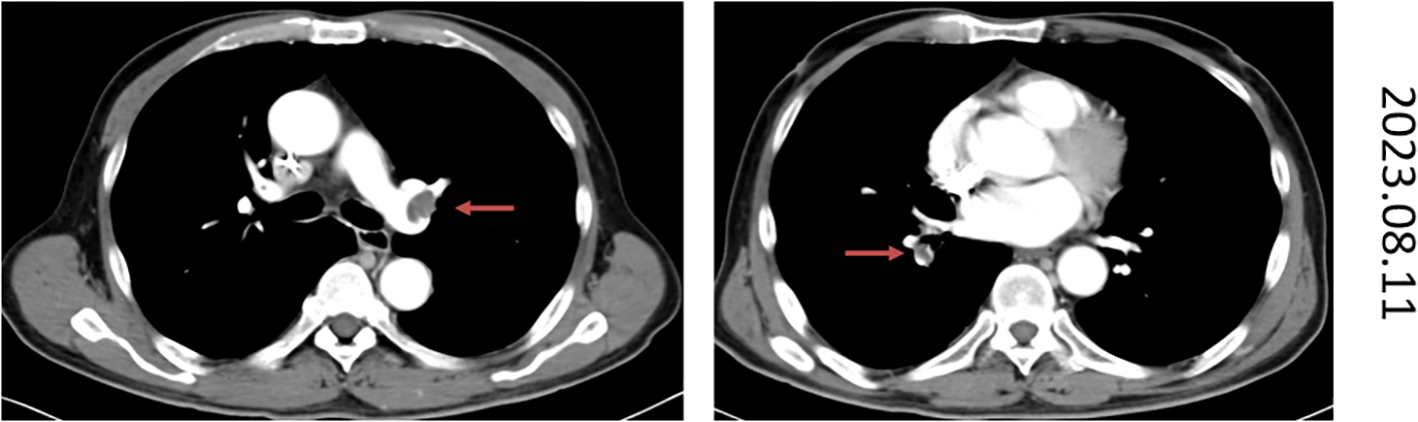
The chest CT image of August 11, 2023 demonstrates typical imaging findings of pulmonary embolism.

The specific treatment plan is as follows: the patient received 13 cycles of mFOLFIRINOX regimen chemotherapy. During the course, maintenance therapy with S-1 was administered for a period. Disease progression (PD) was documented following the final FOLFIRINOX treatment. The date of disease progression (PD) was May 11, 2023 (**Figure 1**). At the same time, the patient’s hemoglobin had decreased to 73 g/dL, and CA199 elevated to 78574 U/ml. The patient’s condition progressed rapidly, the treatment regimen was switched to second-line GA regimen. After two cycles of treatment, the treatment efficacy was also PD. Later, he received the third-line treatment of lenvatinib combined with sintilimab (an anti-PD-1 immune checkpoint inhibitor), and percutaneous hepatic arterial chemoembolization was performed on July 6, 2023. Based on the enhanced CT scan (2023–08–11), the target tumor was evaluated as PD. At the same time, the enhanced chest CT scan showed pulmonary embolism (**Figure 2**), and the lower limb venous color ultrasound showed partial thrombosis in the intermuscular vein and peroneal vein, and received anticoagulation therapy with oral anticoagulants and low-molecular-weight heparin at a dose of 0.6ml every 12 hours.

**Figure 3:**
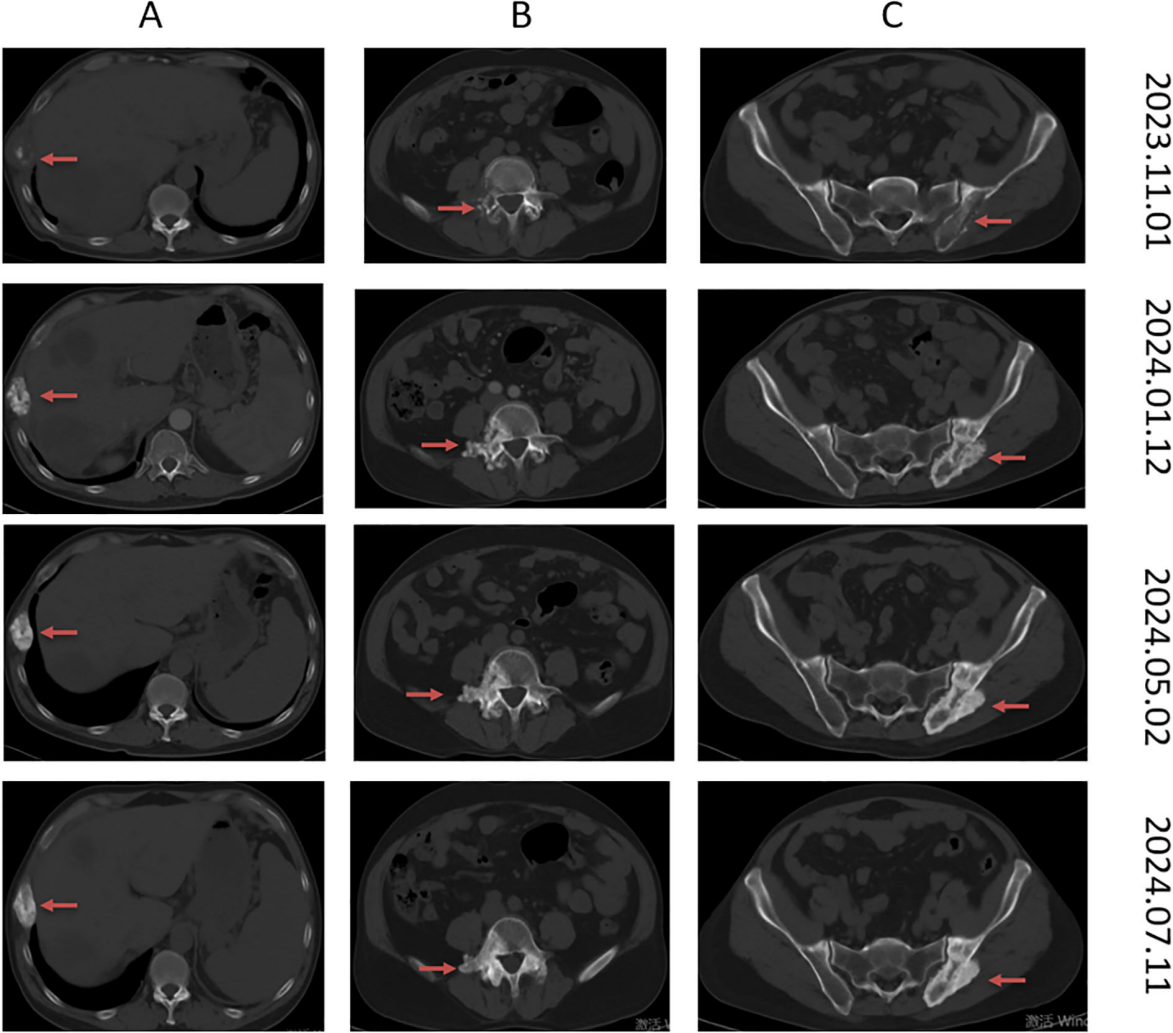
Typical imaging of bone metastasis. The November 1, 2023 imaging revealed bone metastases at the right 8th rib (axillary segment), L4 vertebral body and appendages, left ilium, and right acetabulum. The January 12, 2024 scan was obtained after four cycles of liposomal irinotecan plus fluorouracil regimen. The May 2, 2024 scan was obtained after six months of liposomal irinotecan plus fluorouracil treatment. The July 11, 2024 scan was obtained after completion of 12 cycles of liposomal irinotecan plus fluorouracil therapy followed by three cycles of maintenance treatment with S-1. **(A–C)** demonstrate the changes in metastatic lesions at the right 8th rib (axillary segment), L4 vertebral body and appendages, and left ilium, respectively, before and after treatment. The red arrow points to the tumor.

**Figure 4:**
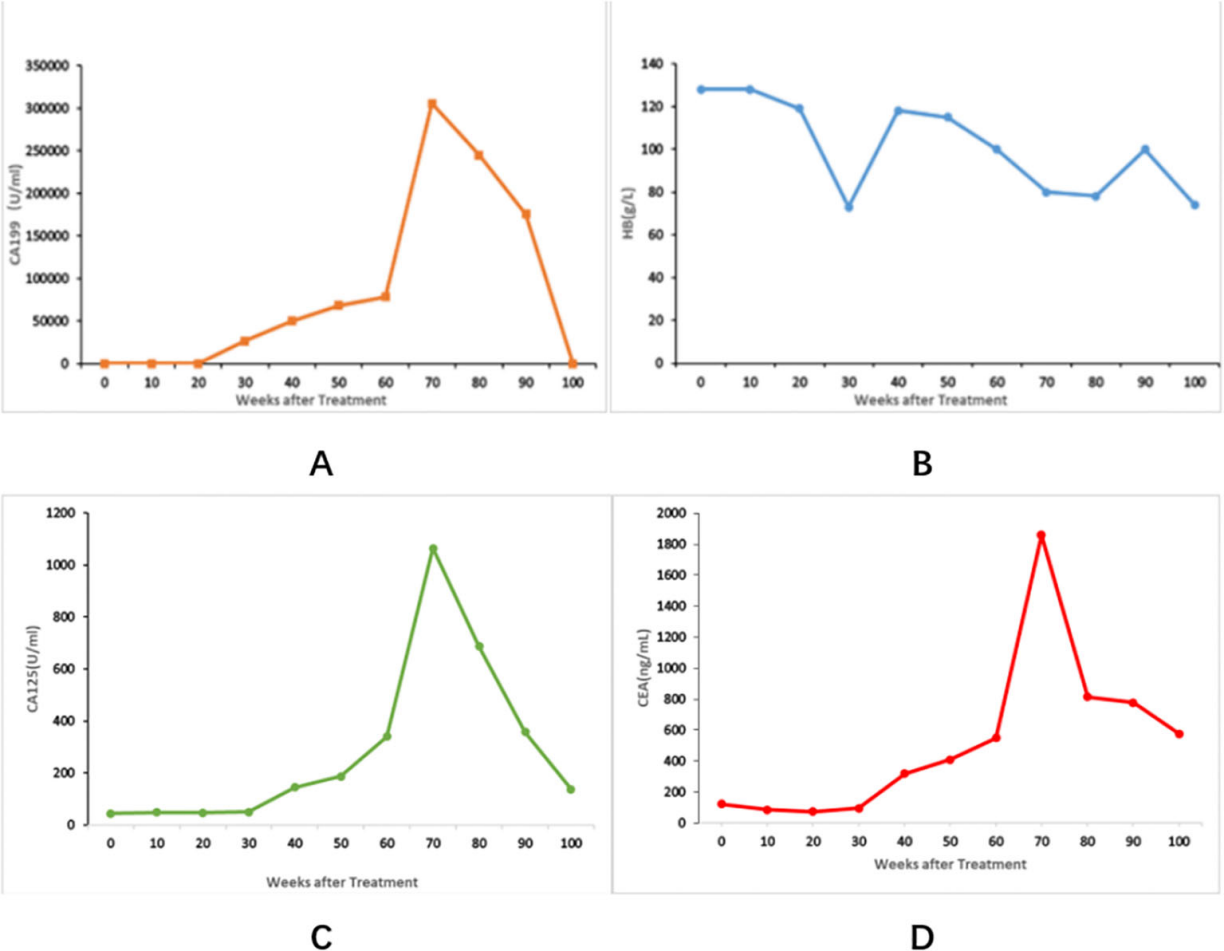
Changes in various indicators during the treatment period. The time zero point on the x-axis of Image 4 represents “weeks after the initiation of anti-tumor therapy for the patient”. The treatment was administered from week 0 to week 100. **(A)** shows the CA19–9 trend. **(B)** shows the hemoglobin (HB) trend. **(C)** shows the CA-125 trend. **(D)** shows the carcinoembryonic antigen (CEA) trend.

During the multi-line treatment process, the patient developed high-risk pulmonary artery thrombosis and lower extremity venous thrombosis, combined with severe anemia. Even though we administered erythropoiesis-stimulating agents and supplemented with granulocyte-colony stimulating factors to manage the anemia symptomatically, the patient still faced a high risk associated with anti-tumor treatment. The patient was recommended to undergo local radiotherapy, but the patient refused and chose to receive oral Chinese medicine treatment. Unfortunately, the enhanced CT scan performed in November 2023 confirmed several new enhanced masses located in the right 8th rib axilla, lumbar vertebrae and appendages, left iliac bone, and right hip socket, accompanied by tissue destruction (**Figure 3**). In addition, tumor marker levels continued to rapidly increase, and the efficacy evaluation was PD. During this period, the patient reported back pain and received symptomatic treatment with 50mg q12h of OxyContin. The pain was controlled. Considering the poor differentiation and rapid progression of malignant tumors, the patient received treatment with liposomal irinotecan, fluorouracil, and calcium folinate. From November 2023, the patient received the following treatment regularly: liposomal irinotecan 86mg ivgtt d1+calcium folinate 600mg ivgtt d1+fluorouracil 1800mg civ24h d1-2, q2w.Two months later (during a total of four cycles of treatment), routine CT scans showed significant reduction in liver and bone lesions; The patient has been undergoing regular follow-up examinations. The CT scan performed on May 2, 2024, after six months of treatment with liposomal irinotecan plus fluorouracil, demonstrated significant shrinkage of the liver metastases. Tumor markers also significantly decreased (**Figure 4**); The qualitative value of CEA decreased from 2339mAU/ml to 82.9 mAU/ml, and the qualitative value of CA-199 decreased from 156904ng/ml to 6806ng/ml, combined with chest and abdominal CT efficacy evaluation of PR.

**Figure 5:**
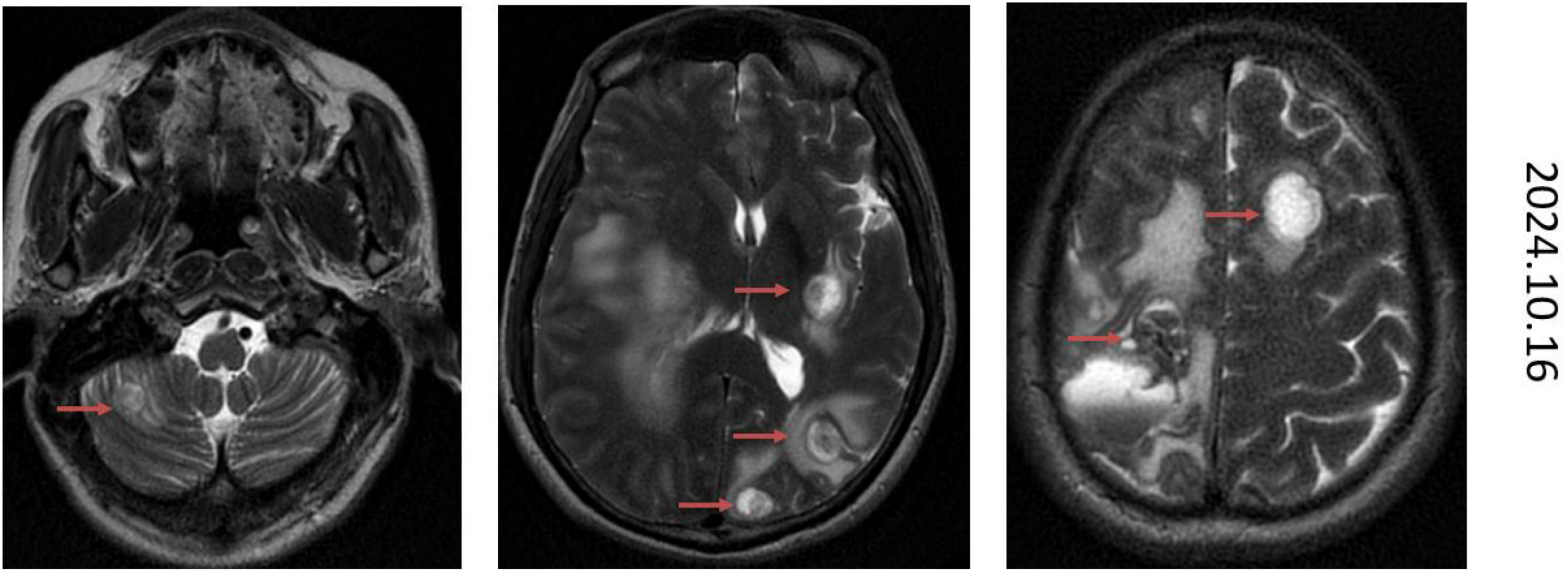
October 16, 2024 contrast-enhanced magnetic resonance imaging (MRI) of the brain demonstrates characteristic radiographic features consistent with cerebral metastases.

**Figure 6:**
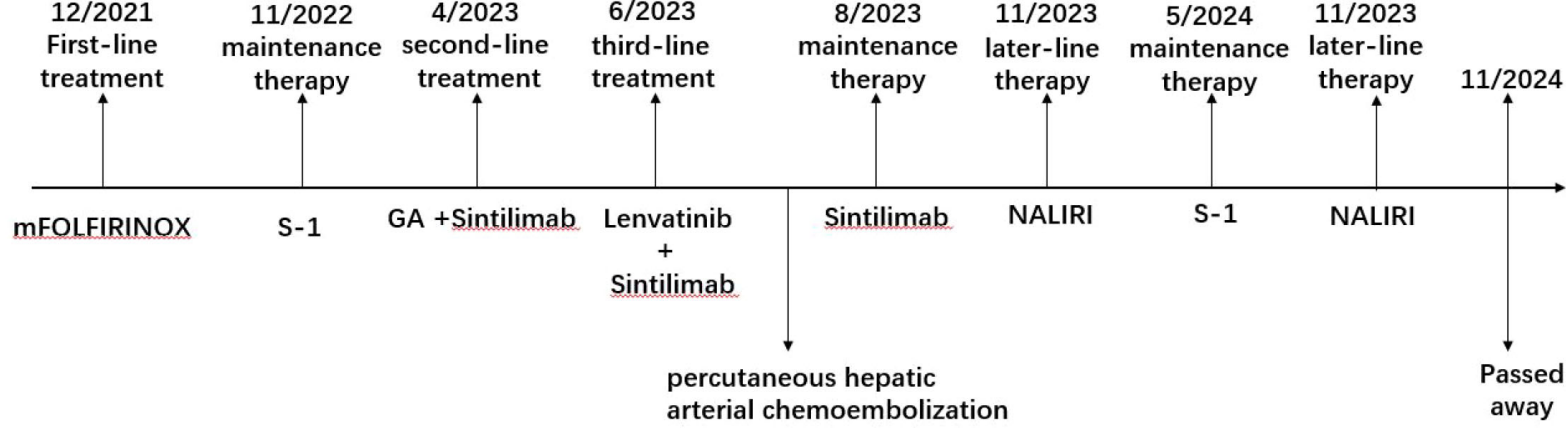
Timeline of patient treatment.

The patient has undergone 12 cycles of NALIRI chemotherapy from 2023-11–15 to 2024-5-6, during which he developed grade 2 anemia, grade 2 platelet decrease, and grade 2 leukocyte decrease, which was well tolerated, and the patient experienced Grade 1 nausea managed with ondansetron and Grade 1 diarrhea. The patient has survived for 12 months after this regimen treatment, and the efficacy evaluation after 4, 8 and 11 cycles is PR, and good treatment results have been achieved with liposomal irinotecan (**Figure 1**). After 12 cycles, oral S-1 60mg bid, d1-d14, q3w maintenance therapy was started, the MRI showed multiple metastases in the head on 2024-10-16 (**Figure 5**) and patient passed away on 2024-11-13 (**Figure 6**).

## 3 Discussion

Pancreatic cancer is known as the most malignant cancer with very poor prognosis. Its global incidence rate has more than doubled in the past 25 years. The diagnosis of pancreatic cancer is quite challenging because most cases are asymptomatic at an early stage. As the disease progresses, non-specific symptoms such as jaundice, weight loss, abdominal pain, and fatigue gradually appear. Patients with locally advanced or metastatic PDAC are often considered incurable and are usually treated with palliative care. How to improve the quality of life of patients and prolong their lives is one of the most concerned issues in the world today. At present, the performance of standard dose gemcitabine and FOLFIRINOX (5-fluorouracil, folinic acid, irinotecan, and oxaliplatin) in advanced PDAC patients has been compared in the PRODIGE 4-ACCORD11 phase III trial. This phase 3 multicenter trial demonstrates that the FOLFIRINOX regimen is a good and effective first-line treatment option, with a median OS of 11.1 months and progression free survival of 6.4 months. Therefore, we chose FOLFIRINOX as the first-line treatment option for the patient. The patient’s condition is still under control at first, and the treatment efficacy is SD. However, after 13 repeated cycles of FOLFIRINOX treatment, the tumor efficacy evaluation was PD. Meanwhile, the tumor marker CA-199 increased significantly, the hemaglobulin decreased to 69g/dL and the patient condition became worse.

Although chemotherapy remains the cornerstone of systemic treatment, options become scarce once first-line therapy fails. Since the emergence of immune checkpoint inhibitors, the immunotherapy has brought significant survival benefits and greatly changed the treatment mode of many malignant tumors. Although immunocheckpoint blocking therapy has shown significant efficacy in the treatment of various types of tumors, it is ineffective in many trials involving pancreatic cancer. Immunotherapy with immune checkpoint blockade (ICB) alone has shown limited efficacy. Combination therapies hold significant potential for enhancing immune responses and achieving better therapeutic outcomes. ICB treatment uses specifically designed antibody drugs (immune checkpoint inhibitors) to block the interaction between checkpoint proteins and their ligands. This releases the tumor-induced suppression of the immune system, allowing immune cells to regain their ability to attack tumor cells (3).TKIs have the potential to modulate the tumor immune microenvironment (TME) by inhibiting angiogenesis, regulating immune cell function, reducing inflammation, and decreasing fibrosis (4). Researchers, based on exploratory analyses of subgroup studies and translational research, have begun to identify signals of PDAC patient subgroups that may potentially benefit from immune checkpoint therapy (IO) or targeted therapy.

Due to the rapid progression of the disease, we have used lenvatinib and sintilimab as a third-line treatment. Besides, the patient received percutaneous hepatic artery chemoembolization on July 6, 2023. Sintilimab is a natural PD-1 inhibitor that binds to PD-1 and blocks its binding to PD-L1 and PD-L2, relieving immunosuppressive effects, activating T cell function, enhancing T cell immune surveillance and killing ability against tumors, and generating tumor immune responses (5). Unfortunately, the efficacy was subsequently assessed as PD.

At this time, the liposome irinotecan (Nal-IRI) was listed in Chinese Mainland. Considering the progress of the disease, the treatment plan was changed to liposome irinotecan+fluorouracil+calcium folinate. Liposomal irinotecan is a type of intravenous liposome preparation that encapsulates the TOP1 inhibitor irinotecan in lipid bilayer vesicles (6). Liposomal irinotecan is composed of a single-layer lipid bilayer vesicle encapsulating irinotecan in the form of sucrose octasulfate colloid or precipitate. PEGylation protects the liposome from recognition by the mononuclear phagocyte system, so liposomal encapsulation of irinotecan maintains a longer circulation time before metabolic conversion to the active metabolite SN-38, thereby improving pharmacokinetic characteristics. After 24 hours of Nal-IRI administration, approximately 95% of the effective payload of irinotecan remains in the liposome, allowing for higher drug loading compared to non-liposome irinotecan. Through rational design, the particle size of liposomal irinotecan is approximately 110 nm, which prevents penetration through normal vascular wall gaps while enabling specific targeting to tumor regions via the enhanced permeability and retention (EPR) effect. This promotes continuous accumulation of the drug in tumor tissues, followed by slow release of the active irinotecan component, ultimately reducing toxicity and enhancing therapeutic efficacy (6–8).In the NAPOLI-1 Phase III trial, mPDAC patients were randomly assigned to receive nal-IRI+5-FU/LV (n=117), nal-IRI (n=151), or 5-FU/LV (n=149) in the first 4 weeks of 6 cycles. The results showed that Nal-IRI+5-FU/LV had a survival benefit compared to 5-FU/LV, and prognostic markers greater than 1 year of survival were identified, including OS, median PFS, and ORR (9). Besides, in the HE072-CSP-004 study focusing on Chinese patients with advanced pancreatic cancer, the NALIRIFOX regimen (liposomal irinotecan + 5-FU/LV + oxaliplatin) significantly prolonged median progression-free survival compared to the AG regimen (gemcitabine + nab-paclitaxel) (7.7 vs. 3.7 months) and showed a trend toward overall survival benefit (12.9 vs. 8.9 months), with an objective response rate of 30.8% and a manageable safety profile (10). Surprisingly, after only two months of treatment, the patient’s liver and bone metastases were well controlled. In addition, the levels of tumor markers rapidly decrease. Although the patient experienced varying degrees of side effects such as anemia and suffered from bone destruction caused by bone metastasis during the treatment process, the patient’s condition was controlled and the efficacy was evaluated as PR. While the NAPOLI-1 clinical trial has demonstrated that the combination of nanoliposome irinotecan (Nal-IRI) with fluorouracil (5-FU)/leucovorin (LV) can prolong survival in patients with metastatic pancreatic ductal adenocarcinoma (PDAC) previously treated with gemcitabine, this particular case presented the following characteristics: disease progression after three prior lines of therapy including 11 cycles of FOLFIRINOX (which contains conventional irinotecan), advanced disease stage, poor performance status, and concurrent pulmonary trunk embolism. Remarkably, treatment with Nal-IRI plus 5-FU/LV rapidly achieved partial response (PR) with progression-free survival (PFS) lasting 10 months, while causing only mild adverse effects and gradual improvement of anemia symptoms. However, we did not systematically document the patient’s quality of life or related treatment experiences throughout the entire course of therapy. This case report showed that Nal-IRI can demonstrate remarkable efficacy even after multiple lines of therapy, offering a valuable treatment option for heavily treated irinotecan resistant patient with advanced pancreatic cancer.

## 4 Conclusion

Despite significant advancements in pancreatic cancer treatment, the prognosis remains extremely poor, with persistently high mortality rates. In this case report, we describe a Chinese male patient diagnosed with advanced pancreatic cancer and liver metastases. The disease progressed rapidly despite first-line FOLFIRINOX chemotherapy, second-line gemcitabine plus albumin-bound paclitaxel (GA), and third-line lenvatinib plus sintilimab. However, after switching to liposomal irinotecan (Nal-IRI) combined with fluorouracil (5-FU) and leucovorin (LV), the patient achieved a partial response (PR), with progression-free survival (PFS) lasting 10 months. These findings suggest that liposomal irinotecan may achieve favorable efficacy in the treatment of pancreatic cancer after multiple lines of therapy, even after the development of irinotecan resistance. However, as a single-case retrospective report, the findings are limited by individual variability, potential biases inherent in retrospective data collection, and the absence of a control group. Therefore, prospective, large-scale clinical studies are warranted to systematically evaluate the efficacy and safety of liposomal irinotecan in patients with irinotecan-resistant advanced pancreatic cancer, thereby further validating its clinical applicability.

## Data Availability

The datasets presented in this study can be found in online repositories. The names of the repository/repositories and accession number(s) can be found in the article/supplementary material.
